# Joint exposure to plasma heavy metals and the risk of kidney graft failure

**DOI:** 10.3389/ti.2026.16919

**Published:** 2026-06-23

**Authors:** Yaqin Yang, Pien Rawee, Mengjie Song, Antonio W. Gomes-Neto, Jacob van den Born, Martin H. De Borst, Daan J. Touw, Stephan J. L. Bakker, Ron T. Gansevoort, Michele F. Eisenga

**Affiliations:** 1 Division of Nephrology, Department of Internal Medicine, University of Groningen, University Medical Center Groningen, Groningen, Netherlands; 2 Department of Clinical Pharmacy and Pharmacology, University of Groningen, University Medical Center Groningen, Groningen, Netherlands

**Keywords:** arsenic, cadmium, heavy metal, kidney graft failure, lead

Dear Editors,

Long-term kidney allograft survival remains a major clinical challenge. Nearly half of deceased-donor transplants fail within a decade, and identifying modifiable risk factors is therefore a priority [1]. Environmental heavy metals such as cadmium (Cd), lead (Pb), and arsenic (As) are established nephrotoxins that promote oxidative stress, tubular injury, and progression of chronic kidney disease [2]. Kidney transplant recipients (KTRs) are particularly susceptible because of reduced renal clearance, comorbidities, and immunosuppressive therapy. Previous work by our group demonstrated that each of these metals independently predicts graft failure [3, 4]. However, real-world exposure involves mixtures rather than single agents, and no prior study has evaluated their combined impact on long-term graft outcomes. We addressed this gap using advanced mixture modeling in a large prospective cohort.

We analyzed 668 outpatient KTRs from the TransplantLines Food and Nutrition Biobank and Cohort Study. Plasma Cd, Pb, and As were quantified by inductively coupled plasma mass spectrometry. A composite mixture index was derived using quantile-based g-computation (Q-gcomp), which jointly estimates the effect of increasing all metals by one quantile while weighting each metal’s contribution [5]. The primary outcome was death-censored graft failure. Bayesian Kernel Machine Regression (BKMR) was applied to capture non-linear and interactive effects, multivariable linear regression identified clinical determinants of the mixture index, and prespecified subgroup analyses were stratified by baseline estimated glomerular filtration rate (eGFR) and proteinuria.

The median age was 54.7 years (IQR 44.8–62.9), 58% of participants were male, and median eGFR was 38.7 mL/min/1.73 m^2^, assessed at a median of 5.3 years post-transplant. Plasma metal concentrations rose significantly across mixture-index tertiles, while inter-metal Spearman correlations between metals were generally weak (all r < 0.4), with distinct concentration distributions across KTRs (Figure 1A), supporting their treatment as distinct yet co-occurring exposures. Higher tertiles were accompanied by lower eGFR (47.4–31.3 mL/min/1.73 m^2^, *P* < 0.001), greater proteinuria, lower transferrin saturation (TSAT), hemoglobin, and iron, and higher high-sensitivity C-reactive protein (hs-CRP) and uric acid, suggesting clustering of metal burden with kidney injury, anemia, and inflammation.

**FIGURE 1:**
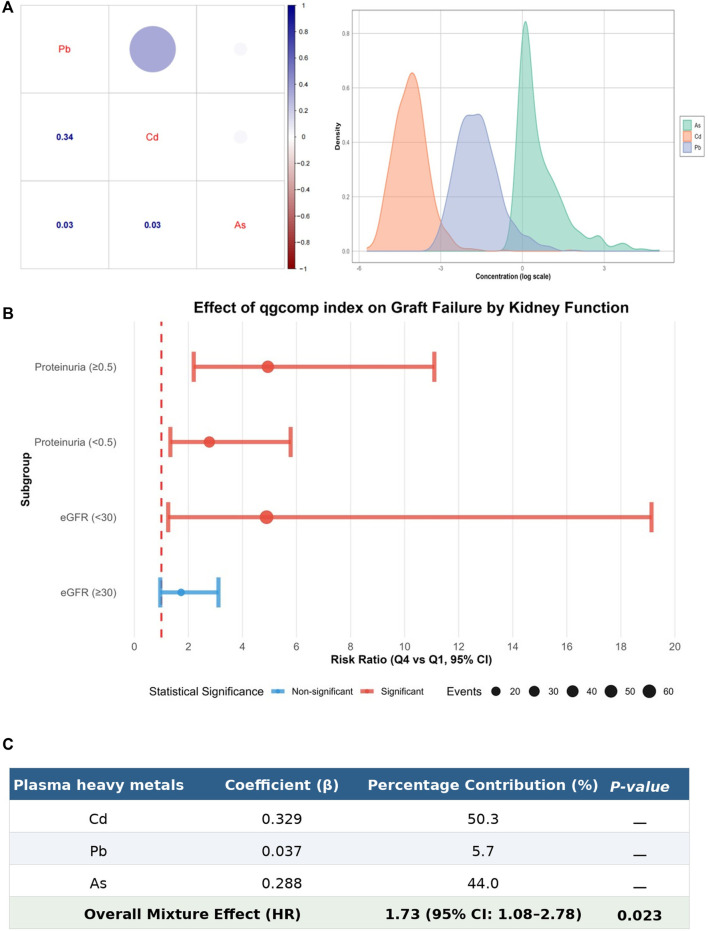
Combined heavy metal exposure and graft failure risk in kidney transplant recipients. **(A)** Correlation matrix and log-scale concentration distributions of plasma Cd, Pb, and As. **(B)** Subgroup analysis of the mixture effect on graft failure stratified by baseline kidney function (risk ratio, Q4 vs. Q1). **(C)** Q-gcomp mixture weights and overall hazard ratio for graft failure.

During a median follow-up of 5.3 (IQR 4.5–6.0) years, 80 graft failure events occurred. In Q-gcomp analysis, a simultaneous one-tertile increase in all three metals was associated with a 73% higher risk of graft failure (HR 1.73, 95% CI 1.08 to 2.78, *P* = 0.023, Figure 1C). Cd contributed 50.3% of the overall mixture effect, As 44.0%, and Pb only 5.7%, identifying Cd and As being the dominant nephrotoxic drivers in this population. For comparison, individual metal associations with graft failure are presented in Supplementary Results (Supplementary Figure S1), which showed consistent patterns with Cd and As independently associated with increased risk. BKMR analysis confirmed a dose-dependent increase in risk above the 50th exposure percentile and revealed a synergistic interaction in which the nephrotoxic effect of Cd was amplified at higher As concentrations (Supplementary Figure S2). Multivariable regression further identified higher eGFR (β = −0.260, *P* < 0.001), longer transplant duration, and higher TSAT as inverse correlates of mixture burden, whereas higher fish intake was a positive correlate, pointing to clinically actionable determinants of systemic exposure.

A key observation was the strong variation in metal-associated graft failure risk according to baseline kidney function. Among KTRs with severe renal impairment (eGFR below 30 mL/min/1.73 m^2^ or proteinuria of at least 0.5 g/24 h), those in the highest quartile of metal exposure had nearly a fivefold increased risk of graft failure compared with the lowest quartile (RR 4.89, 95% CI 1.25 to 19.12, and RR 4.87, 95% CI 2.10 to 11.30, respectively, Figure 1B). In contrast, the risk difference was much smaller and only borderline significant among recipients with preserved eGFR (RR 1.74, 95% CI 0.96–3.16) or low-grade proteinuria (RR 2.79, 95% CI 1.35–5.77). This gradient likely reflects impaired metal clearance and increased tissue accumulation in damaged kidneys, compounded by elevated oxidative stress and inflammation that sensitize the kidney to metal toxicity [6, 7]. These findings support incorporating baseline kidney function into risk assessment strategies for KTRs and identify a high-risk subgroup that may benefit most from targeted intervention.

From an environmental perspective, our findings align with concerns that current regulations, which typically evaluate metals individually, may underestimate risks from combined exposures. Epidemiological data from CKD-endemic regions such as Nicaragua and Sri Lanka show that agricultural workers chronically exposed to mixed metals develop kidney injury even at low levels [8]. Our results extend these observations to a vulnerable transplant population in a high-income setting, indicating that the burden of mixed exposure is not confined to occupationally or geographically high-risk groups, and underscoring the need for safety standards that explicitly address metal mixtures.

The inverse association between TSAT and the mixture index suggests a protective effect of adequate iron status, as iron and cadmium compete for shared transporters such as DMT1 and ZIP proteins, affecting both intestinal absorption and tissue retention [9]. This finding is consistent with our previous report of an inverse relationship between TSAT and cadmium levels in KTRs and underscores the bidirectional interplay between iron metabolism and metal toxicity in this population [9]. The positive association with fish intake likely reflects dietary exposure to bioaccumulated environmental contaminants, particularly inorganic As and Cd in long-lived predatory species. Beyond lifestyle modification, chelation therapies such as ethylenediaminetetraacetic acid (EDTA) have proven effective in lowering circulating metals and slowing renal decline in other chronic conditions, raising the possibility of benefit in selected KTRs with elevated mixture indices [10]. Limitations include reliance on a single baseline plasma measurement, the observational design that precludes causal inference, and limited generalizability beyond similar outpatient cohorts.

In conclusion, this prospective study establishes that combined heavy metal exposure significantly increases graft failure risk in KTRs, with Cd and As demonstrating synergistic nephrotoxic effects. Importantly, we identified that recipients with compromised kidney function are particularly vulnerable to metal-induced graft injury, suggesting the need for risk-stratified clinical management. These findings support integrating routine heavy metal monitoring into post-transplant care, especially for high-risk recipients, which may help mitigate metal-associated graft loss and improve long-term transplant outcomes.

## Data Availability

The data analyzed in this study is subject to the following licenses/restrictions: Access to the data can be requested through the University Medical Center Groningen. Requests to access these datasets should be directed to Michele F. Eisenga, m.f.eisenga@umcg.nl.
