# Supplementary material for: Joint exposure to plasma heavy metals and the risk of kidney graft failure
Source: Transpl Int. 2026 Jun 23;39:16919. doi: 10.3389/ti.2026.16919 (PMC13337517; doi:10.3389/ti.2026.16919)
Supplement: Supplementary file 1 [file Supplementaryfile1.docx]

**Online Supplementary Material**

**Joint Exposure to Plasma Heavy Metals and the Risk of Kidney Graft Failure**

Yaqin Yang1, M.D., Pien Rawee♯1, MSc, Mengjie Song♯1, MSc, Antonio W Gomes-Neto1, M.D., PhD, Jacob van den Born1, PhD, Martin H De Borst1, M.D., PhD, Daan J Touw2, PharmD, PhD, Stephan J L Bakker1, M.D., PhD, Ron T Gansevoort1, M.D., PhD, FERA, FASN, Michele F Eisenga1, M.D., PhD*

*1Division of Nephrology, Department of Internal Medicine, University of Groningen, University Medical Center Groningen, Groningen, The Netherlands.*

*2Department of Clinical Pharmacy and Pharmacology, University of Groningen, University Medical Center Groningen, Groningen, The Netherlands*

*♯ Co-second authorship*

**Corresponding author**

M.F. Eisenga, M.D., PhD

Department of Internal Medicine, Division of Nephrology

University Medical Center Groningen

P.O. Box 30.001, 9700 RB Groningen, the Netherlands

Phone: 0031 050 361 61 61, Email: [m.f.eisenga@umcg.nl](mailto:m.f.eisenga@umcg.nl)

**Supplementary Results**

**Association of individual plasma metals with risk of graft failure**

Results from Cox regression models are illustrated in **Figure S1**. No significant association was observed between plasma lead concentrations and graft failure in any of the models. In contrast, higher plasma concentrations of cadmium and arsenic were independently associated with an increased risk of graft failure.

For cadmium, the crude model demonstrated a significant association (HR 1.88, 95% CI: 1.46-2.41, *P* < 0.001). This association remained consistent after adjusting for demographic and clinical transplant-related factors in Adjusted Model 1 (HR 1.87, 95% CI: 1.46-2.38, *P* < 0.001). In the fully adjusted model (Adjusted Model 2), each log-transformed ng/L increase in plasma cadmium increase in plasma cadmium was associated with a 53% higher risk of graft failure (HR 1.53, 95% CI: 1.10-2.15, *P* = 0.013).

Similarly, for arsenic, the crude model indicated a significant association (HR 1.24, 95% CI: 1.01-1.54, *P* = 0.044), which strengthened in Adjusted Model 1 (HR 1.36, 95% CI: 1.09-1.71, *P* = 0.008). In the fully adjusted model (Adjusted Model 2), each log-transformed ng/L increase in plasma cadmium increase in plasma arsenic was associated with a 32% higher risk of graft failure (HR 1.32, 95% CI: 1.03-1.71, *P* = 0.031).

**Supplementary Figures**

**Figure S1.** Associations of log-transformed heavy plasma metals with graft failure risk.


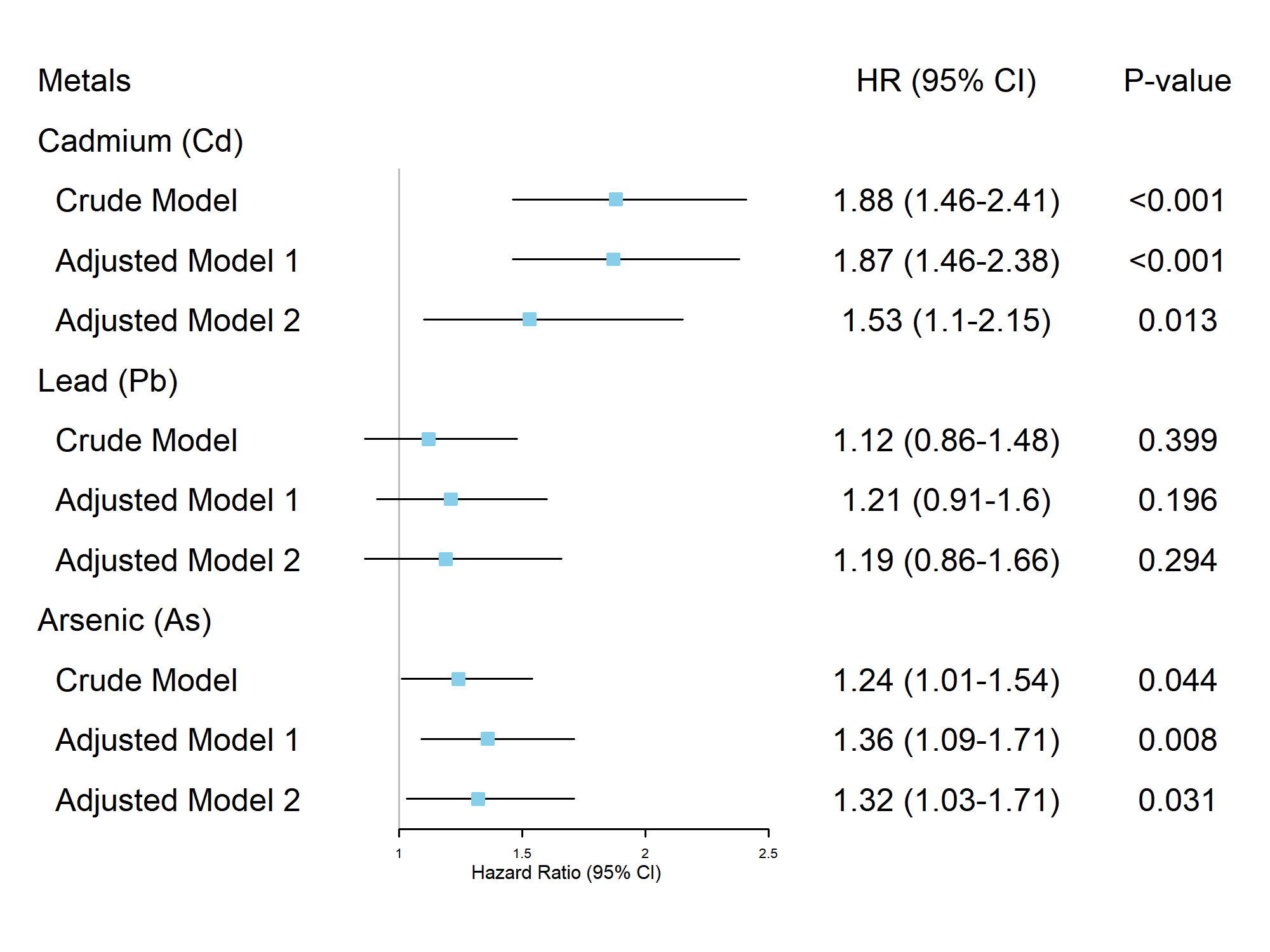


Notes: Crude model, without adjustment. Adjusted model 1, adjusted for demographic and clinical transplant-related factors; Adjusted model 2, adjusted for laboratory indices, medication use and kidney function indicators.

**Figure S2.** The associations of plasma heavy metal mixtures and graft failure risk evaluated by BKMR model.


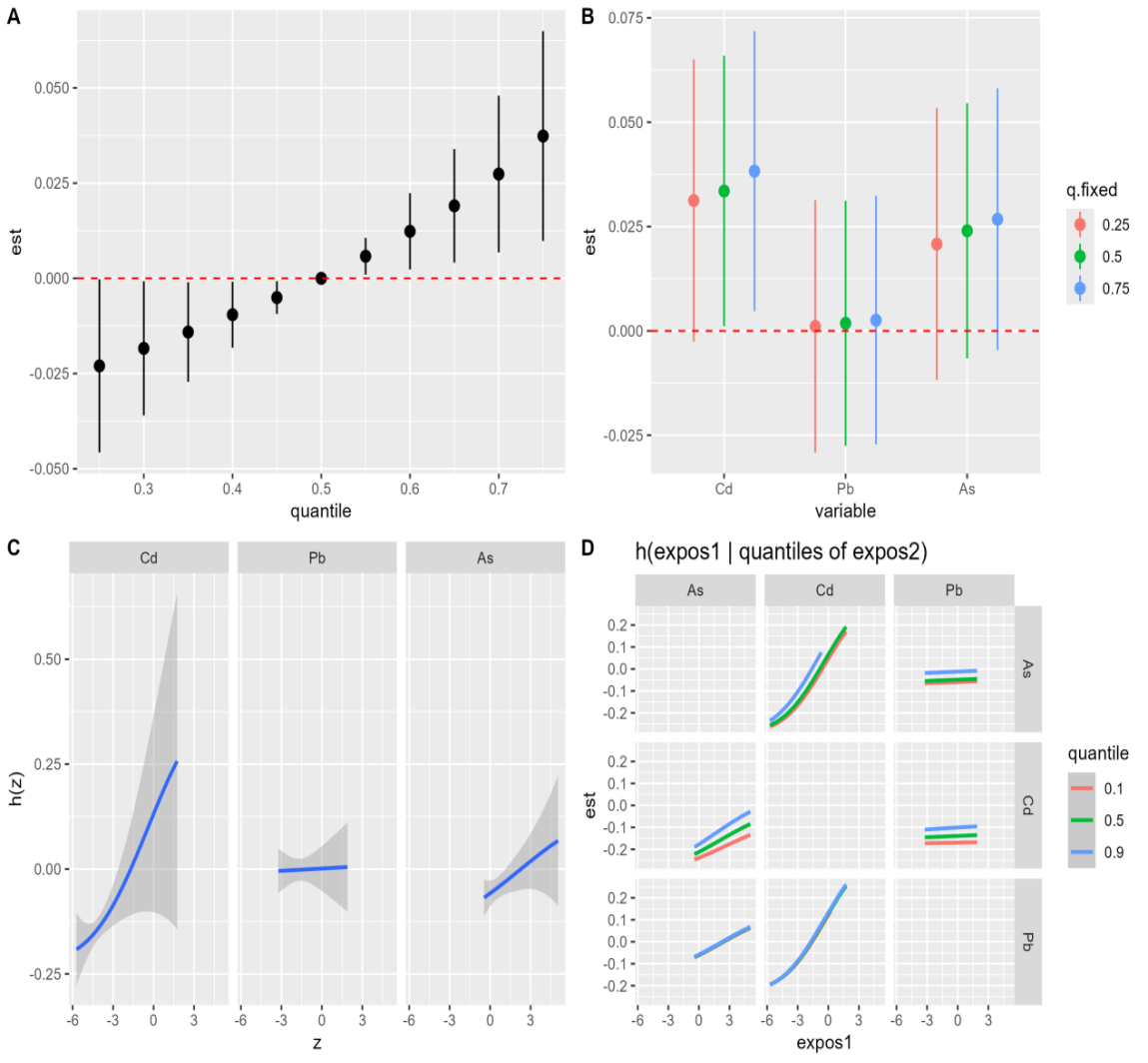


(A) The overall joint effects of heavy metal mixtures on graft failure risk were evaluated using BKMR. The analysis compared the risk associated with all metals at specified percentiles to a baseline risk where all metals were set at their median (50th percentile). (B) The associations between individual heavy metals and graft failure risk were assessed using BKMR, with the concentrations of other metals held constant at the 25th (red), 50th (green), or 75th (blue) percentiles. (C) Exposure-response relationships between individual heavy metals and graft failure risk were visualized using BKMR. (D) Interaction effects between heavy metals were explored using BKMR. All BKMR models were adjusted for demographic and clinical transplant-related factors, laboratory indices, medication use, and kidney function indicators.
